# Lipid Rafts, Sphingolipids, and Ergosterol in Yeast Vacuole Fusion and Maturation

**DOI:** 10.3389/fcell.2020.00539

**Published:** 2020-07-03

**Authors:** Logan R. Hurst, Rutilio A. Fratti

**Affiliations:** ^1^Department of Biochemistry, University of Illinois at Urbana-Champaign, Urbana, IL, United States; ^2^Center for Biophysics and Quantitative Biology, University of Illinois at Urbana-Champaign, Urbana, IL, United States

**Keywords:** membrane fusion, sphingolipids, membrane trafficking, lipid rafts, vacuole

## Abstract

The *Saccharomyces cerevisiae* lysosome-like vacuole is a useful model for studying membrane fusion events and organelle maturation processes utilized by all eukaryotes. The vacuolar membrane is capable of forming micrometer and nanometer scale domains that can be visualized using microscopic techniques and segregate into regions with surprisingly distinct lipid and protein compositions. These lipid raft domains are liquid-ordered (L_*o*_) like regions that are rich in sphingolipids, phospholipids with saturated acyl chains, and ergosterol. Recent studies have shown that these lipid rafts contain an enrichment of many different proteins that function in essential activities such as nutrient transport, organelle contact, membrane trafficking, and homotypic fusion, suggesting that they are biologically relevant regions within the vacuole membrane. Here, we discuss recent developments and the current understanding of sphingolipid and ergosterol function at the vacuole, the composition and function of lipid rafts at this organelle and how the distinct lipid and protein composition of these regions facilitates the biological processes outlined above.

## Introduction

The lysosomal vacuole of *S. cerevisiae* plays a number of important roles in cellular homeostasis. The importance of the eukaryotic lysosomal compartment is underscored by the fact that there are over 70 human diseases whose molecular basis is intricately tied to dysfunctions of lysosomal storage and homeostasis (Platt et al., 2018). The fungal vacuole is necessary for nutrient storage, protein turnover, and detoxification, but it also plays a large and often-underappreciated role in essential processes such as autophagy, ion homeostasis, and organelle contact and fusion (Li and Kane, 2009). Membrane fusion is a conserved biological process that occurs at most, if not all, cellular membranes, and is necessary for autophagy, secretion and endocytic recycling. The process of homotypic vacuolar fusion in yeast cells is essential for maintaining osmotic balance, pH balance, and vacuolar inheritance by the daughter cell, and this system has proven to be instrumental in understanding many of the basic mechanisms of membrane fusion (Wickner, 2010). Through years of exemplary work by a number of labs across the world many of the factors that are necessary for fusion have been identified, and in most cases a molecular mechanism for these effectors has been investigated in detail. These fusion effectors include proteins such as tethers, Rab/Rho GTPases, SNAREs, chaperones and actin, and the regulatory lipids phosphatidic acid (PA), diacylglycerol (DAG), ergosterol, and phosphatidylinositol phosphates (PtdInsPs), which have been discussed in recent review articles (Wickner and Rizo, 2017; Langemeyer et al., 2018; Starr and Fratti, 2019; Ungermann and Kümmel, 2019). The specific lipid composition of cellular membranes is recognized as a key factor in the multitude of essential biological processes performed in or on these membranes (Harayama and Riezman, 2018). The complex lipid composition of cellular membranes has been suggested to be a driving force behind the formation of regions of phase separation and lateral heterogeneities known as “lipid rafts,” whose biological significance is only just beginning to be understood (Sezgin et al., 2017). While there are detailed molecular mechanisms that describe glycerophospholipids as regulatory lipids in homotypic fusion, there is a lack of such research focusing on the role that sphingolipids play in this process, even though some species of these lipids were found to be enriched in endosomal and lysosomal fractions in earlier studies (Hechtberger et al., 1994). It has become abundantly clear that sphingolipids play a large role in lysosomal function and homeostasis in humans, providing ample reason to investigate the molecular mechanism(s) for sphingolipid-based regulation of lysosomal processes in other models (Kihara, 2016; Hannun and Obeid, 2018). In this review we highlight research that has focused on this subject and discuss outstanding questions and future perspectives related to it.

## Sphingolipids in Membrane Trafficking and Endosomal Maturation

Membrane trafficking and maturation events have been extremely well-studied using the yeast system as a model over the past 40 years, and many of the pathways and effectors have been identified and investigated at varying levels (Feyder et al., 2015). Defects in vesicular trafficking have been identified in a number of studies concerning yeast impaired for sphingolipid synthesis and turnover. The yeast ortholog of human neutral sphingomyelinase type 2, Isc1p, catalyzes the removal of the headgroup sugars from glycosylated sphingolipids. Yeast that lack Isc1p are unable to degrade complex sphingolipids, causing a buildup of mannose-inositol-phosphoceramide (MIPC) and M(IP)_2_C, as well as altered levels of the second messenger long chain bases (LCBs) and LCB-1-phosphates (Sawai et al., 2000; Barbosa et al., 2011). These yeast also produce more non-hydroxylated C26-ceramide species, and less α-OH-phytoceramide species, presumably from an increase in *de novo* ceramide synthesis (Barbosa et al., 2011). In *isc1Δ* yeast, lysosomal trafficking is significantly altered, evidenced by the secretion of vacuole-destined carboxypeptidase Y (CPY/Prc1p) into the extracellular space, which could not be rescued by overexpression of Vam3p or Ykt6p and was attributed to increased ceramide activated protein phosphatase (CAPP) activity (Teixeira et al., 2016). Such a large increase in non-hydroxylated ceramide species as seen in the studies referenced above could have a thickening effect on cellular membranes, reducing the lateral mobility and fluidity of the bilayer and preventing the proper organization of effectors necessary for maturation and fusion. Defects in ceramide homeostasis and subsequent downstream signaling events were also detected in yeast with a temperature sensitive *SEC14* allele and those that lack the endosomal SNARE Tlg2p (Mousley et al., 2008). The block in endosomal maturation resulted in widespread changes to the sphingolipid profile of these strains. The vacuolar SNAREs that promote the docking step of fusion were improperly trafficked when sphingolipid synthesis was disrupted by depletion of the acyl-CoA-binding protein Acb1p, and vacuoles from these strains were unable to fuse (Faergeman et al., 2004). Furthermore, work with the fission yeast *S. pombe* highlighted a role for MIPC in the trafficking of proteins to the plasma membrane and proper vacuole homeostasis (Nakase et al., 2010). These studies demonstrate the intimate ties between endosomal maturation and sphingolipid biosynthesis and turnover in yeast.

Endosomal maturation is a complex process that intertwines anterograde trafficking from *trans*-Golgi network (TGN) derived vesicles with endocytosed material from the cell’s exterior, ultimately to be degraded, recycled and/or sent to different organelles (Huotari and Helenius, 2011). By utilizing HPLC and advanced mass spectrometry techniques it was discovered that wild-type yeast sphingolipids have much less variation in acyl chain length and saturation compared to mammalian sphingolipids, almost exclusively requiring a saturated C26 very long chain fatty acid (VLCFA) bound via amide link to a LCB (Lester et al., 1993; Ejsing et al., 2006, 2009; Megyeri et al., 2016). This C26 VLCFA requirement is reinforced by the lethality of attempting to knockout *ELO2* (formerly FEN1) and *ELO3* (formerly SUR4) simultaneously (Revardel et al., 1995). These membrane-embedded, ER-localized proteins catalyze the first step of microsomal VLCFA elongation through the condensation of a long chain fatty acid with a malonyl-CoA unit (Denic and Weissman, 2007). A high-temperature suppressor screen revealed that increased expression of the endosomal Rab GTPase Vps21p could rescue the growth defect observed in *elo3Δ* yeast, and *elo3Δvps21Δ*, *elo3Δvps3Δ*, *elo3Δvps19Δ*, and *elo3Δvps8Δ* strains had significantly impaired growth rates (Obara et al., 2013). The proteins Vps3p/Vps8p are subunits of the CORVET endosomal-tethering complex, and Vps19p (Vac1p/Pep7p) is a PtdIns3P binding protein that functions in many steps of membrane trafficking (Weisman and Wickner, 1992; Webb et al., 1997; Plemel et al., 2011). The authors observed a similar genetic interaction by creating a *csg1Δcsh1Δvps21Δ* triple mutant strain, which blocked *de novo* synthesis of MIPC instead of disrupting VLCFA production. This reinforced the idea that the endosomal maturation process is dependent on C26-sphingolipids and the endosomal fusion machinery. The *elo3Δvps21Δ* yeast also showed abnormal proteolytic and glycosylation/maturation profiles for CPY, Pep4p (Proteinase A), Pho8p (alkaline phosphatase), and Cps1p (carboxypeptidase S/CPS). The Snc family of SNARE proteins participates in exocytic, endocytic, and recycling processes, and their function is regulated by complex sphingolipids, VLCFAs, and LCB-1-phosphates (David et al., 1998; Grote et al., 2000; Tani and Kuge, 2012). Taken together, these studies strongly suggest that sphingolipids play an active role in the membrane maturation events at endosomes and lysosomes in yeast.

## Ergosterol and Raft Domains

The vertex ring domain that forms between docked vacuoles is enriched in proteins and lipids that are necessary for fusion, including ergosterol (Wang et al., 2002; Fratti et al., 2004; Karunakaran et al., 2012). Ergosterol has also been shown to be necessary for the activation of Cdc42p at the vacuole, promoting homotypic fusion that coincides with, but is not dependent on, Sec18p/Sec17p priming activity (Jones et al., 2010). Although it was shown that ergosterol production was necessary for activation, it’s unknown if this is due to physical interactions between GTPase and sterol, lipid raft modulation, or even through modulating the activity of a guanine nucleotide exchange factor (GEF) or GTPase activating protein (GAP) for Rho proteins at the vacuole.

Ergosterol and sphingolipids in yeast are key players in the lipid raft hypothesis. Liquid phase separation of lipids into stable micrometer domains was observed in giant unilamellar vesicles (GUVs) when monitoring the temperature- and lipid composition-dependent distribution of a fluorescent lipid-based dye (Veatch and Keller, 2003). These coexisting liquid phases were determined to be a liquid-disordered (L_*d*_) like phase consisting of unsaturated acyl-chain phospholipids and a L_*o*_ like phase composed of saturated acyl-chain phospholipids and cholesterol. Therefore, it wasn’t entirely surprising that the same phenomenon could also be observed with GUVs made from isolated yeast PtdInsPs (containing an unsaturated acyl chain), ergosterol, and inositol-phosphoceramide (IPC; saturated acyl chains) (Klose et al., 2010). This study utilized Laurdan spectroscopy experiments to measure the anisotropy and movement of the dye within the membrane, and the results suggested that the formation of these domains depended on the presence of C26-phytosphingolipid species due their disappearance from GUVs when the lipids were isolated from *elo3Δ* or *sur2Δ* yeast. Some legitimate criticisms and questions about the presence of these membrane microdomains arose from their seemingly short lifetimes *in vivo*, invasive techniques for isolation and microscopic observation, and physiological relevance of phase transition temperatures. However, many of these questions were addressed when micrometer-scale membrane domains were visualized in live yeast for the first time with relatively non-invasive visualization techniques (Toulmay and Prinz, 2013). This study showed that selected vacuolar proteins with a fluorescent tag could segregate into one of two distinct and separate domains when yeast cells are grown to stationary phase. This segregation could also be induced by glucose starvation, translation inhibition via cyclohexamide, or mild acidic stress, indicating they likely have biological relevance in vacuole homeostasis. Interestingly, two proteins highlighted in this study, Vph1p (discussed below) and Ivy1p, have known and putative roles in the homotypic vacuole fusion cascade, respectively. Ivy1p is a possible I-BAR protein with a role in sensing membrane curvature for fusion and/or fission processes, and is known to interact with the Rab GTPase Ypt7p and the HOPS subunit Vps33p, both of which are necessary for the tethering step of vacuole fusion (Lazar et al., 2002; Numrich et al., 2015; Itoh et al., 2016). Ivy1p was shown to colocalize with the macrolide filipin III that binds to ergosterol in yeast vacuoles, indicating that Ivy1p resides in L_*o*_ like rafts enriched in ergosterol and sphingolipids following domain formation in the vacuole membrane. A cursory investigation of the proteins that were found to reside in the L_*d*_ like domain in the seminal study revealed that they all contain multiple transmembrane helices. Contrary to this, the proteins that localized to the L_*o*_ like domains are soluble proteins that interact with the vacuole membrane via larger membrane complexes (Gtr2p tethers to the membrane via the EGO complex, and Ivy1p binds to Ypt7p and HOPS complex), implying there may yet be a correlation between a protein’s structural biology and its residential membrane domain, but this will require in-depth studies. A screen for yeast mutants that were unable to produce these lipid raft domains in vacuoles pointed to genes in phospholipid production (*NEM1* and *FAB1*) and endosomal maturation/multivesicular body (MVB) formation (*VPS4*) (Toulmay and Prinz, 2013). There have been some recent advances in understanding how these domains may form in the vacuolar membrane. Tsuji and colleagues showed that the yeast Niemann-Pick type C (NPC) orthologs Ncr1p and Npc2p are essential components for the creation and growth of the L_*o*_ ergosterol rich domain (Tsuji et al., 2017). This study also highlighted that that MVB pathway at the endosome facilitates ergosterol transport to the vacuole, again highlighting the interconnectivity of membrane trafficking and homeostasis mechanisms in yeast.

## V-ATPase Function and Localization

The vacuolar ATPase (V-ATPase) is required for proper acidification of the organelle, which is necessary for the activation of resident hydrolases and autophagy, and Vph1p (V_0_ subunit) forms *trans*-complexes between vacuoles that promote homotypic fusion, and interacts with a number of fusion effectors (Nakamura et al., 1997; Peters et al., 2001). It was also shown that the localization of Vph1p can shift to the boundary under alkaline conditions for turnover via intralumenal fragment (ILF) recycling (McNally et al., 2017). Studies have linked the activity of the V-ATPase to sphingolipid levels in the vacuole membrane. It was discovered that *isc1Δ* yeast are unable to acidify the vacuole, and this was due to increased Sit4p-mediated CAPP activity (Teixeira et al., 2016). The V_1_ component of the V-ATPase requires VLCFAs for proper activity and is regulated by Orm1/2p controlled serine-palmitoyl transferase activity (Chung et al., 2003; Finnigan et al., 2011). Conversely, it was shown that the loss of individual V-ATPase components can drastically alter the levels of complex sphingolipids and hydroxylated species, indicative of a functional relationship between these lipids and vacuolar acidification (Tani and Toume, 2015). As previously mentioned, Vph1p was shown to segregate into a specific membrane compartment that is separate and distinct from the L_*o*_ like region enriched in Ivy1p and ergosterol (Toulmay and Prinz, 2013). This Vph1p-containing region was determined to be L_*d*_ like in nature due to an observed colocalization with the marker dye Fast DiI. The proton translocation activity is modulated via the regulatory lipid phosphatidylinositol 3,5-bisphosphate (PtdIns(3,5)P_2_), which would historically correlate well with a L_*d*_ membrane domain (Li et al., 2014; Banerjee et al., 2019). There is at least one study that challenges the model of PtdIns(3,5)P_2_ distributing to the L_*d*_ like region and suggests a pool may also enrich in the L_*o*_ like membrane rafts. This research used a quick-freezing and freeze-fracture replica labeling (QF-FRL) electron microscopy (EM) method coupled with a highly specific PtdIns(3,5)P_2_ binding probe to show that a large fraction of this lipid in the vacuole localizes to Ivy1p-enriched/Vph1p-depleted region following hyperosmotic stress (Takatori et al., 2016). This could suggest that there is a pool of PtdIns(3,5)P_2_ that is remodeled or synthesized with saturated acyl chains, and that membrane microdomains can be induced by distinct mechanisms possibly leading to multiple raft species with distinct lipid and protein compositions.

**FIGURE 1:**
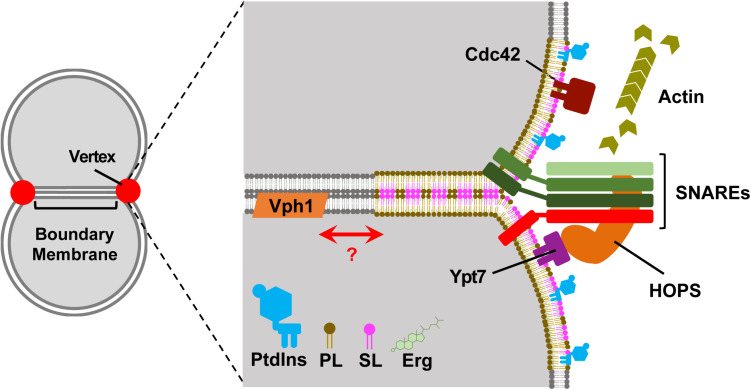
The vertex domains of docked vacuoles may represent another lipid raft species. Left, Two-dimensional representation of docked vacuoles. Upon docking vacuoles form distinct morphological regions. The boundary membrane is the flattened area of apposed membranes. The vertex domains form at the transition between the boundary and outer undocked membranes. Right, A zoomed in view of the vertex-boundary transition area. The membranes are composed of disordered and ordered membrane raft-like microdomains. The disordered regions are composed of generalized phospholipids (e.g., phosphatidylcholine) in gray. The vertex domain is a raft-like domain shown as being thicker and contains sphingolipids (SL, pink), ergosterol (Erg, green), phosphoinositides (PtdIns, blue), and vertex-localized phospholipids (PL, mocha). The vertex domain contains SNAREs, Cdc42, Ypt7 and recruits the HOPS complex and is a site for actin polymerization. The V-ATPase subunit Vph1 is present in the ordered and disordered regions of the membrane, but fully assembled and functional V-ATPase requires *de novo* sphingolipid synthesis (not shown).

It is tempting to relate the location of Vph1p and Ivy1p membrane domains to the location and topography of the fusion machinery between tethered and docked vacuoles. We know that ergosterol, Ypt7p, and the HOPS complex localize to the vertex region during fusion, and Vph1p is normally mostly found ubiquitously throughout the vacuole membrane (Wang et al., 2002; Fratti et al., 2004; McNally et al., 2017). It is possible that the vertex region enriched in ergosterol is another L_*o*_ like microdomain, which may even form with a different lipid/protein composition than those identified above (Figure 1). Vacuoles are constantly undergoing fission and fusion events when cells are early in their chronological lifespan, which implies the membrane microdomains that only form after reaching stationary phase when vesicular trafficking has decreased would likely have less impact on the vacuolar fission/fusion process. We now know that lipid raft domains can form in the vacuole following a number of stress inputs (reviewed in Tsuji and Fujimoto, 2018), and it will require diligent work to piece together the contributing pathways and factors for the formation different domains. It would be interesting to see if the observable phase separation of vacuole membrane regions can persist following isolation on floatation gradients, and whether it can be induced with changes in pH or osmotic pressure.

## Autophagy

The vacuole is necessary for the highly conserved and essential autophagy degradation pathway that delivers cytoplasmic components for hydrolysis and recycling. This process is highly regulated by a number of mechanisms, including nutrient levels and target of rapamycin complex 1 (TORC1) activity at the vacuole (Reggiori and Ungermann, 2017). It was found that this process is also intimately tied to sphingolipids as autophagic flux and aminopeptidase I (Ape1p) processing are defective in *isc1Δ* yeast, and this could be rescued by downregulating the TORC1-Sch9p pathway (Teixeira et al., 2016). It is notable that the TORC1 stimulating GTPase Gtr2p of the vacuolar EGO complex localized to L_*o*_ like membrane rafts, adding another possible layer of autophagic communication between sphingolipid-rich lipid rafts and TORC1 signaling (Toulmay and Prinz, 2013). It was also shown that blocking endosomal maturation could lead to altered sphingolipid homeostasis and increased ceramide levels, which was found to block the unfolded protein response (UPR) at the ER and TORC1 activity, and these effects were again tied to Sit4p CAPP activity (Mousley et al., 2008). When yeast reach stationary phase they produce more lipid droplets (LDs) presumably to maintain lipid homeostasis within the cell (Walther and Farese, 2012). Wang and colleagues showed that the L_*o*_ like raft domains in the vacuole mediate the autophagy of these organelles (lipophagy), and this is dependent on a subset of canonical autophagy related genes (Atg) (Wang et al., 2014). Together these studies confirm the presence of a complicated relationship between sphingolipid metabolism and autophagy whose intricacies remain to be completely understood.

## Conclusion and Future Perspectives

Most sphingolipid biosynthetic genes display varying levels of interactions with genes annotated as having a function in membrane trafficking and endolysosomal maturation. According to the *Saccharomyces* Genome Database^1^ *AUR1* (the catalytic subunit to IPC synthase) displays genetic interactions with *ATG1*, *GYP1*, *PEP1*, *SEC17*, *VAM7*, *VPH1*, *VPS4*, *VPS8*, *VPS9*, *VPS13*, *VPS21*, *VPS41*, and *VPS55*. Future studies will require clever and well-planned *in vitro* and *in vivo* studies to understand the full contribution of sphingolipids and lipid rafts in the fusion cascade. Although complex sphingolipids are likely to be trafficked to the vacuole within the lumenal leaflet because their biosynthetic enzymes are housed in the lumen of the Golgi, ceramide may face either leaflet due to it higher rate of bilayer translocation or flip-flop, and there may be yet unknown or non-canonical transfer methods to move sphingolipids between leaflets (Levine et al., 2000; Sato et al., 2009). Alternatively, it is possible that the C26 VLCFA facilitates clustering across leaflets, as has been shown for GPI-linked proteins at the plasma membrane (Raghupathy et al., 2015). It would be ideal to create reconstituted systems to examine the sphingolipid contribution to the fusion mechanism, such as those done with SNAREs and proteoliposomes (Zick et al., 2014). Reconstituted *in vitro* systems will require careful isolation and purification of yeast sphingolipids due to a lack of commercial availability, but they will allow for tight control of the system to observe the direct contribution of individual sphingolipids to membrane fusion. It is likely that both L_*o*_ and L_*d*_ like membrane domains play a role in vacuole fusion, with each being enriched in different regulatory lipids and proteins that promote fusion. This is supported by the observation that the vertex ring domains are not homogeneous in nature and show a distribution of known fusion factors (Wang et al., 2002; Fratti et al., 2004; Wickner, 2010). There is also a desire for a sensitive sphingolipid-binding probe similar to the fluorescently tagged PtdInsP-binding domains that helped researchers determine the localization of PtdInsPs in a living cell (Burd and Emr, 1998; Várnai and Balla, 1998). Yeast will produce C6-NBD-IPC from C6-NBD-ceramide, but they won’t modify it further. This indicates that either substrate recognition for subsequent glycosylation enzymes heavily relies on the presence of a VLCFA bound at the amide position, or the presence of the fluorescent group prevents association with a substrate-binding pocket, and a similar phenomenon has been observed in some mammalian systems (Lipsky and Pagano, 1985; Levine et al., 2000).

While there is much left to discover, studies that focused on understanding the composition and biological relevance of lipid raft domains at the yeast vacuole have provided exciting results. Future studies should strive for technical coverage by utilizing a number of established methods in conjunction. These studies will need to seamlessly mix the cell biology and physical biochemistry of the membrane by using techniques such as mass spectrometry, Laurdan anisotropy, genetic manipulation, and advanced microscopy to name a few. While many studies referenced above use some of these techniques, further advancement in the field will require more thoughtful and advanced *in vitro* and *in vivo* techniques to overcome limitations and obtain a complete picture detailing how the lipid and protein composition of the yeast vacuole changes following stresses and upon reaching stationary phase, and the homeostatic pathways that communicate with this dynamic organelle.

## Author Contributions

LH and RF conceived and wrote the manuscript. All authors contributed to the article and approved the submitted version.

## Conflict of Interest

The authors declare that the research was conducted in the absence of any commercial or financial relationships that could be construed as a potential conflict of interest.

## Footnotes

1 www.yeastgenome.org
